# Investigating the Ethical and Data Governance Issues of Artificial Intelligence in Surgery: Protocol for a Delphi Study

**DOI:** 10.2196/26552

**Published:** 2021-02-22

**Authors:** Kyle Lam, Fahad M Iqbal, Sanjay Purkayastha, James M Kinross

**Affiliations:** 1 Imperial College London London United Kingdom

**Keywords:** artificial intelligence, digital surgery, Delphi, ethics, data governance, digital technology, operating room, surgery

## Abstract

**Background:**

The rapid uptake of digital technology into the operating room has the potential to improve patient outcomes, increase efficiency of the use of operating rooms, and allow surgeons to progress quickly up learning curves. These technologies are, however, dependent on huge amounts of data, and the consequences of their mismanagement are significant. While the field of artificial intelligence ethics is able to provide a broad framework for those designing and implementing these technologies into the operating room, there is a need to determine and address the ethical and data governance challenges of using digital technology in this unique environment.

**Objective:**

The objectives of this study are to define the term digital surgery and gain expert consensus on the key ethical and data governance issues, barriers, and future research goals of the use of artificial intelligence in surgery.

**Methods:**

Experts from the fields of surgery, ethics and law, policy, artificial intelligence, and industry will be invited to participate in a 4-round consensus Delphi exercise. In the first round, participants will supply free-text responses across 4 key domains: ethics, data governance, barriers, and future research goals. They will also be asked to provide their understanding of the term digital surgery. In subsequent rounds, statements will be grouped, and participants will be asked to rate the importance of each issue on a 9-point Likert scale ranging from 1 (not at all important) to 9 (critically important). Consensus is defined a priori as a score of 7 to 9 by 70% of respondents and 1 to 3 by less than 30% of respondents. A final online meeting round will be held to discuss inclusion of statements and draft a consensus document.

**Results:**

Full ethical approval has been obtained for the study by the local research ethics committee at Imperial College, London (20IC6136). We anticipate round 1 to commence in January 2021.

**Conclusions:**

The results of this study will define the term digital surgery, identify the key issues and barriers, and shape future research in this area.

**International Registered Report Identifier (IRRID):**

PRR1-10.2196/26552

## Introduction

The emergence of huge datasets ranging from imaging, sensors, and electronic medical records has resulted in the rapid uptake of artificially intelligent technology across health care [1]. The operating room is no exception; it incorporates digital technologies ranging from augmented reality systems [2] to next generation robotics [3]. The hope of this armory of technology at the surgeon’s disposal is for more efficient, safe, and precise surgery that will in turn improve patient outcomes, lead to more efficient utility of operating theaters, and allow surgeons to progress rapidly up learning curves. Driven by the promise of such rich rewards, uptake in digital technology in the operating theater has been rapid, and this has been further accelerated by the emergence of COVID-19, which has seen widespread adoption of digital technology across health care [4].

However, incorporation of this technology into the operating room is not without risk. Digital systems are inherently dependent on data to function, and the accessing, sharing, and use of huge amounts of potentially sensitive personalized data pose significant risk. Lessons can be learned from the failure of implementation of digital technologies across health care that have been widely reported in the media [5,6]. The risks of mismanagement of these large datasets are often overlooked in the pursuit of furthering efficiency. Therefore, when failures do occur, the net result is the reduction of public trust and ultimately the hindering of development of these technologies. It is therefore vital that we address the key ethical and data governance issues of the transformation into a digital operating room.

We can seek guidance from the implementation of artificial intelligence (AI) across different industries. The field of AI ethics is a response to the potential harms that AI systems can cause such as bias and discrimination, invasion of privacy, and poor-quality outcomes. AI ethics can be defined as “a set of values, principles, and techniques that employ widely accepted standards of right and wrong to guide moral conduct in the development and use of AI technologies” [7]. The 4 key pillars named by the Alan Turing Institute concerning the design and use of AI systems are fairness, accountability, sustainability, and transparency.

While this provides a basis for clinicians and technologists to adhere to for surgical AI systems, the operating room is a unique environment that poses its own specific challenges. Surgical AI systems must contend with issues of consent if future digital systems are dependent on opaque algorithms. There is also the issue not only of privacy of patients but of future surgical teams who will be potentially under scrutiny for every action they take. Questions around litigation and liability are, to date, untested. Finally, not only will the digital operating room be dependent on data, but it has the potential to become a priceless data pipeline leading to issues of data ownership and the potential consequences of commercial partnerships. There is now a critical need to address these ethical and data regulation issues in this digital surgery era.

The objectives of this study are to conduct a Delphi exercise to determine opinions and gain consensus on the key ethical and data regulation issues concerning the use of AI in surgery. Through this process, we will define the term digital surgery and its components and develop a consensus-based list of issues, barriers, and future research goals from a variety of stakeholders across digital surgery.

## Methods

### Justification for Study Design

Delphi exercises have been used widely across health care to determine consensus across a wide variety of issues, and their merits are amplified in areas where there is uncertainty or limited knowledge [8,9]. It is an iterative process of sequential questionnaires designed to combine expert opinion into group consensus [10]. A series of questionnaires are answered and submitted. Following each round of questionnaires, participants receive a summary of the entire panel’s answers from the previous round and are asked to repeat the questionnaire. Participants are encouraged to review the panel responses and revise their own responses and through this process converge toward consensus.

The Delphi technique has several key advantages over face-to-face roundtable discussions. It allows all panelists to be heard equally without domination of a single voice. The feedback mechanism, where results of the panel are returned to participants, also permits participants to change their minds easily having reviewed the views of the rest of the panel [11,12]. Most importantly, however, the Delphi technique has gained popularity for practical reasons: it allows experts to participate all over the world without restriction and therefore is a pragmatic and cost-effective means of gaining consensus.

The Delphi technique, however, is not without criticism. It is reliant on the continued participation of panelists through the rounds of the exercise. As such, Delphi exercises may suffer from a decline in response rate, and this has been a frequent criticism of the Delphi technique [13]. Therefore, efforts must be made to encourage continued participation to prevent attrition bias. In addition, criticisms have been made concerning the reliability of the Delphi technique. Critics have argued there is no guarantee the same results will be obtained should the same information be presented to 2 different panels of experts [13]. It is therefore important to ensure that the panel consists of a broad and diverse representation of experts. Despite these criticisms, however, the Delphi technique continues to be a popular, easy, and low-cost means of determining consensus.

Moreover, the Delphi methodology has been shown to be effective in areas of research where there is uncertainty or little knowledge [13]. There has been, to our knowledge, little work performed in this area. The Delphi methodology is not only a tool for gaining consensus but is also an effective means for idea generation. Novel ideas can subsequently be fed back to the panelists, which will encourage further ideas to be synthesized.

Finally, participation in the Delphi process has been shown to be a highly motivating experience for participants. The feedback mechanism can also be a stimulating process for those engaging in the Delphi process [9]. We hope that this will spark future discussion in this relatively unknown field.

### Ethics

Ethical approval for this study was granted by the local research ethics committee at Imperial College, London (20IC6136). All participants will be required to provide informed consent to take part at the start of the online questionnaire. Data will be handled in accordance with UK data protection regulations.

### Structure

**Figure 1:**
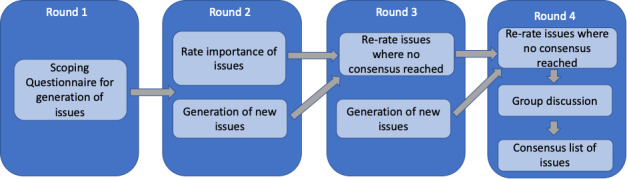
Structure of the Delphi exercise.

The Delphi exercise will consist of 3 online questionnaire rounds and a final live online consensus meeting (Figure 1). Round 1 will consist of an online scoping questionnaire, rounds 2 and 3 will consist of online questionnaires where statements will be rated, and round 4 will take the form of a live online meeting delivered through videoconferencing software. Online questionnaire rounds will be undertaken through Qualtrics software (Qualtrics) and will be active for periods of up to 4 weeks. Those who do not complete the questionnaire will be sent a reminder email weekly.

All invited participants will receive a personalized invitation to participate via email. This will include an explanatory statement about the Delphi exercise, why they have been chosen, and a link to the questionnaire. Participants will be encouraged to complete all rounds, as attrition bias can lead to overestimation of the degree of consensus in the final results [11]. Strategies that we will employ to prevent attrition bias include sending potential participants a personalized pre-Delphi invitation to participate in the first round and listing only those who complete the entire Delphi process in the final publication.

Participants will be quasi-anonymous for online questionnaire rounds; identities of the participants will not be known to other participants but will be known to the study organizers. Anonymity allows equal opportunity for all participants to provide and react to ideas unbiased by the identities of others [13]. While anonymity cannot be achieved in the final online meeting, the result tally of participant votes will be anonymized.

An initial scoping round will encourage panelists to generate statements for subsequent rounds. These statements will be presented to panelists in the two subsequent rounds where the panelists will vote on the importance of the statements; group discussion of the statements will occur at a final online meeting. Through the 4 rounds of the Delphi, we aim first to gain consensus agreement on the term digital surgery and the key components of digital surgery. Second, we aim to identify high value statements across 4 domains within this theme: ethical issues, data governance issues, barriers, and future research goals.

### Selection of International Experts

Due to the broad nature of the subject matter, we aim to recruit experts across multiple sectors: clinical, ethics and law, policy, AI, and industry. These are all key stakeholders in the development and implementation of digital surgery, and involvement of all these sectors is vital to gain a representative view of the key issues. We identified experts as those with national and international profiles in their respective fields, authors of impactful research in the literature, major digital technology companies, and experts recommended by peers. There are no strict exclusion criteria. All individuals identified or recommended by peers as suitable to participate in the Delphi exercise will be sent an initial personalized pre-Delphi invitation email requesting participants to express their interest.

A sample size calculation dependent on statistical power to generate a number of participants required was not calculated. Because the Delphi exercise is dependent more upon gaining a representative view across multiple disciplines, there is no consensus on the minimum number of participants required [12]. We aim to recruit a minimum sample size of 20 across these varying areas, which has been shown to provide reliable and effective judgement [14].

### Round 1

The initial round of the Delphi exercise acts to generate ideas to discover issues relating to the topic of study. It is therefore vital to ask open questions in this round and not impose the study team’s views on the participants thereby introducing bias into the study. Providing a list of potential issues to study participants may subconsciously emphasize the significance of certain issues favorable to the study team rather than those important to the experts undertaking the Delphi exercise [11].

In the first round, we ask participants open questions across 4 key domains: data governance, ethics, barriers, and future research goals. In addition, participants will be asked their understanding of the term digital surgery.

**Figure 2:**
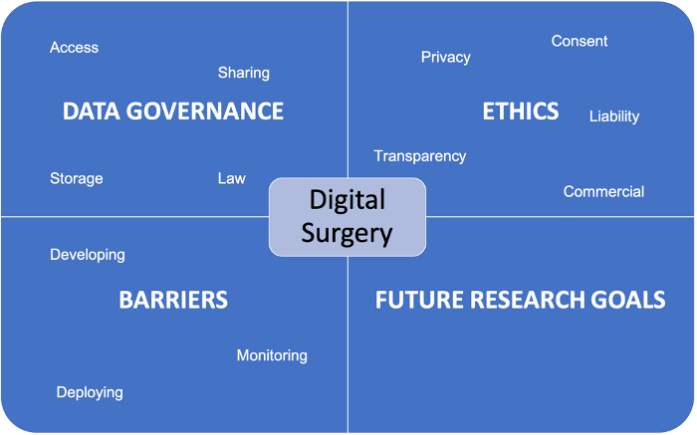
Key domains of round 1.

To facilitate responses, each domain will be further divided into areas we have identified from the literature (Figure 2). We believe this will give more structure and guidance in their free-text answers. In addition, participants will be free to suggest additional issues that may not be covered by our questions.

Laypeople with no technical expertise in any of the associated fields will also be invited to participate in round 1. A version of the questionnaire, understandable to the general public, will be presented to a broad sample of the public. The statements generated by the public will also be presented to the expert panel in round 2.

### Rounds 2 and 3

Only those who complete all previous rounds will be invited to participate in subsequent rounds of the Delphi exercise. Statements from round 1 will be grouped according to common themes and presented to participants alongside statements generated from laypeople with no technical expertise. In both of these online questionnaire rounds, participants will be asked to rank the importance of items according to a 9-point Likert scale, where 1 indicates not important and 9 indicates critical. Issue scoring: 1 to 3 indicates the issue is of little importance, 4 to 6 indicate an issue is important but not critical, and 7 to 9 indicates the issue is critically important. There is no standardized definition of consensus in Delphi exercises. Therefore, we have elected to define consensus as being where the issue is scored 7 to 9 by 70% of respondents and 1 to 3 by less than 30% of respondents, a popular approach used in Delphi exercises [15]. Statements that fail to reach the threshold of consensus will be put forward to the next round of the Delphi consensus process. Participants will also be encouraged to contribute further statements during each of these rounds.

### Round 4

Only participants who have completed round 3 will be invited to participate in round 4. This final consensus meeting will consist of the study team alongside all participants who have completed all previous rounds. The use of a final online consensus meeting was chosen as it facilitates expert interaction in the final round and allows participants to justify their viewpoints and seek further clarification on statements; this has been thought to improve on the original Delphi method [16]. The primary objective of this online meeting is to develop a consensual draft of statements from the Delphi exercise. The meeting will be structured around the nominal group technique, a highly structured group interaction framework [17]. After initial introduction and an explanation of the aims of the meeting, the results of round 3 will be presented to the participants alongside summary descriptive statistics. All nonconsensus statements and newly generated statements from round 3 will first be put forward for voting on the same Likert scale. The definition of consensus will be as per the previous round. All panelist responses will be analyzed together.

Members of the meeting will then be asked to discuss the inclusion and exclusion of statements generated from the entire voting process. Participants will also be encouraged to clarify or further discuss any statements generated. The meeting will be facilitated by members of the study team to ensure all members of the meeting have an equal opportunity to express their views and the discussion is not dominated by a single member. The final consensus statement will be distributed to all those who complete the full Delphi exercise for final approval. Key methodological criteria for the study is detailed in Table 1.

**Table 1:** Key methodological criteria for reporting of Delphi studies as per Diamond et al [18].

Criteria			Response
**Objective**			
	Does the Delphi study aim to address consensus or to quantify level of agreement?	Consensus	
**Participants**			
	How will participants be selected or excluded?	Experts will be from the fields of surgery, artificial intelligence, policy, ethics, and industry. Laypeople will also be asked to respond to a nontechnical version of the scoping questionnaire	
**Methodology**			
	Level of anonymity	Anonymous to other panel members in online questionnaire rounds	
	A priori definition of consensus	Between 7 and 9 on a 9-point Likert scale of importance by 70% of respondents and between 1 and 3 by less than 30% of respondents	
	Criteria used to determine when to stop the Delphi in the absence of consensus?	4 rounds will be conducted in total	

## Results

Infrastructure support for this research was provided by the National Institute for Health Research Imperial Biomedical Research Center. We anticipate round 1 to commence in January 2021 and all Delphi rounds to be completed by Fall 2021. We expect that the study will be published in a peer-reviewed journal and presented at national and international conferences.

## Discussion

### Summary

While the popularity of the use of AI in surgery has increased, there is still a relative paucity of knowledge of the ethical and data governance issues concerning its use. The Delphi exercise described in this paper aims to determine the key issues to be addressed and therefore shape the direction of future research. We hope this work will increase awareness of these issues across all key stakeholders in digital surgery with the ultimate goal of creating not only efficient but ethical surgical AI.

### Strengths and Limitations

The strengths of this study center around the involvement of participants across multiple areas of expertise. This Delphi exercise aims to capture the representative views across clinicians who may use these digital technologies; technologists creating them; and experts in ethics, policy, and law who are concerned with the regulations governing them. We will also include the views of laypeople with no expertise in the fields. It is important to understand the views of the public as digital technology in the operating room is ultimately developed for patient benefit, and therefore public acceptability is of paramount importance. While we aim to recruit participants with an active interest in this field, the study is limited by the willingness of those invited to participate. As such, while we aim to capture a representative sample of all those involved in digital surgery, the views of the experts who participate in the Delphi may differ from those who decline to participate. In addition, we acknowledge that due to the international scope of this study, the use of a final online meeting may limit the attendance of panelists from differing time zones. We believe this is outweighed, however, by the benefits of a live online meeting that will allow clarification and debate of statements. The hosting of two separate meetings to facilitate differing time zones can also be considered should this be required.

### Conclusion

This paper describes the protocol of a Delphi consensus exercise that will aim to define the term digital surgery and identify the key ethical and data governance issues, barriers, and future research goals of the use of AI in surgery. The results of this study will shape future research in this area.

## Abbreviations

AI: artificial intelligence
